# Core–Shell‐Heterostructured Magnetic–Plasmonic Nanoassemblies with Highly Retained Magnetic–Plasmonic Activities for Ultrasensitive Bioanalysis in Complex Matrix

**DOI:** 10.1002/advs.201902433

**Published:** 2019-12-13

**Authors:** Liangwen Hao, Yuankui Leng, Lifeng Zeng, Xirui Chen, Jing Chen, Hong Duan, Xiaolin Huang, Yonghua Xiong, Xiaoyuan Chen

**Affiliations:** ^1^ State Key Laboratory of Food Science and Technology School of Food Science and Technology Jiangxi Key Laboratory for Microscale Interdisciplinary Study Nanchang University Nanchang 330047 P. R. China; ^2^ The People's Hospital in Jiangxi Province Nanchang 330006 P. R. China; ^3^ Laboratory of Molecular Imaging and Nanomedicine (LOMIN) National Institute of Biomedical Imaging and Bioengineering (NIBIB) National Institutes of Health (NIH) Bethesda MD 20892 USA

**Keywords:** core–shell heterostructures, highly retained magnetic‐plasmonic activities, lateral flow immunoassays, magnetic–plasmonic nanoassemblies, self‐assembly

## Abstract

Herein, a facile self‐assembly strategy for coassembling oleic acid‐coated iron oxide nanoparticles (OC‐IONPs) with oleylamine‐coated gold nanoparticles (OA‐AuNPs) to form colloidal magnetic–plasmonic nanoassemblies (MPNAs) is reported. The resultant MPNAs exhibit a typical core–shell heterostructure comprising aggregated OA‐AuNPs as a plasmonic core surrounded by an assembled magnetic shell of OC‐IONPs. Owing to the high loading of OA‐AuNPs and reasonable spatial distribution of OC‐IONPs, the resultant MPNAs exhibit highly retained magnetic–plasmonic activities simultaneously. Using the intrinsic dual functionality of MPNAs as a magnetic separator and a plasmonic signal transducer, it is demonstrated that the assembled MPNAs can achieve the simultaneous magnetic manipulation and optical detection on the lateral flow immunoassay platform after surface functionalization with recognition molecules. In conclusion, the core–shell‐heterostructured MPNAs can serve as a nanoanalytical platform for the separation and concentration of target compounds from complex biological samples using magnetic properties and simultaneous optical sensing using plasmonic properties.

## Graphical abstract

Herein, the facile synthesis of magnetic–plasmonic nanoassemblies (MPNAs) is reported, which exhibit a typical core–shell structure, wherein oleylamine‐coated gold nanoparticles (OA‐AuNPs) preferentially aggregate and form a plasmonic core and oleic acid‐coated iron oxide nanoparticles (OC‐IONPs) assemble a magnetic shell. The resultant MPNAs hold the highly retained magnetic–plasmonic activities for the separation and simultaneous optical sensing of target compounds in complex biological samples.

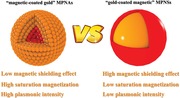

## 1 Introduction

Multifunctional nanoparticles (NPs) are emerging composite NPs that comprise more than one component and have recently attracted considerable interest due to their wide‐ranging applications in imaging,^1^ sensing,^2^ therapy,^3^ catalysis,^4^ and separation.^5^ Among the available multifunctional NPs, nanomaterials with magnetic and plasmonic dual components are promising because they possess intrinsic optical and magnetic properties to support various functions, including biolabeling, bioimaging, bioanalysis, and bioseparation.^6^ Ideal magnetic–plasmonic nanostructures (MPNSs) should possess strong magnetic response and excellent plasmonic signal transducers. In recent years, various strategies have been designed to fabricate diverse MPNSs by growing Au shell or attaching isolated Au nanoparticles (AuNPs) onto the surface of iron oxide NPs (IONPs).[qv: 6c,d,7] The obtained nanocomposites exhibit a typical core–shell “gold‐coated magnetic” nanostructure. However, the saturation magnetization of such nanostructures dramatically decreases with the increase in the plasmonic component because of the inherent magnetic shielding effect of Au shell deposited on the IONPs' surface.^8^ Although this dilemma can be partially alleviated by reducing the plasmonic components, the corresponding plasmonic activities are largely compromised. To our best knowledge, the rational design of MPNSs that can simultaneously maximize the saturation magnetization and the plasmonic optical activity still remains a huge challenge. Thus, a simple and versatile synthetic strategy for fabricating a desirable nanoarchitecture that allows the magnetic and plasmonic components to achieve the harmonious integration inside individual MPNSs is urgently needed.

Theoretically, when magnetic components are used as the shell of the plasmonic materials to form a novel “magnetic‐coated gold” core–shell‐heterostructured nanocomposite, the magnetic response can be retained because the inherent magnetic properties of magnetic components cannot be shielded by the plasmonic components. In addition, the plasmonic optical activity in such MPNSs can be readily improved by increasing the mass percent of the plasmonic components. Thus, precisely controlling the distribution of AuNPs and IONPs in MPNSs is critical for realizing the directed self‐assembly of oleylamine‐coated gold nanoparticles (OA‐AuNPs) into a plasmonic core and oleic acid‐coated iron oxide nanoparticles (OC‐IONPs) into a uniformly distributed magnetic shell to construct the desired “magnetic‐coated gold” core–shell‐heterostructured nanomaterials. Recently, size segregation,^9^ entropy‐driven,^10^ surface charge,^11^ and chemical polarity^12^ strategies have been developed to modulate the phase separation of different NP components in multifunctional nanomaterials and achieve the ordered and directed NP distribution for incessantly improving the physicochemical and optical properties of composite nanomaterials. For example, Chen et al. designed magnetofluorescent core–shell super‐nanoparticles with obvious phase separation of IONPs assembling into a magnetic core and CdSe/CdS quantum dots (QDs) assembling into a fluorescent shell, thus causing significantly enhanced photoluminescence.^12^ In addition, several representative MPNSs with phase‐separated structures such as yolk–shell magnetic–plasmonic nanohybrids^13^ and dumbbell‐like magnetic–plasmonic dimers^14^ were designed and fabricated to minimize the multicomponent interference for optimized magnetic and plasmonic activities. However, the precise synthesis of such MPNSs is complicated and depends on elaborate molecular design.

Self‐assembly based colloidal chemistry synthesis routes have been recognized as a versatile approach for preparing various heterostructured nanocrystals with unique architectures and multicomponent spatial distributions.^15^ In this case, the phase separation of different NP components can be affected not only by the NP surface properties (e.g., surface wettability, charge, chemical group, and energy) but also by the NP's morphology and size, as well as the reactive solvent system.^16^ Among various self‐assembly systems, the directed self‐assembly of different NP components mediated by polymer compatibility has attracted wide interest in the field of multifunctional material fabrication because of its convenience and simplicity.^17^ Our previous work reported the successful self‐assembly synthesis of core–shell‐heterostructured magnetic‐fluorescent nanobeads (MFNBs), in which poly(maleicanhydride‐*alt*‐1‐octadecene) (PMAO) and poly(methyl methacrylate) (PMMA) were introduced to induce the phase separation of oleylamine‐coated CdSe/ZnS QDs (OA‐QDs) and OC‐IONPs on the basis of the solubility difference of OA‐QDs and OC‐IONPs in the polymer matrices of PMAO and PMMA.^18^ The results indicated that OA‐QDs and OC‐IONPs were mainly distributed in the outer layer of MFNBs. Such rational spatial distribution of OA‐QDs and OC‐IONPs in MFNBs caused remarkably enhanced luminescence and effectively retained the saturation magnetization.

In this study, we report the facile synthesis of magnetic–plasmonic nanoassemblies (MPNAs) by coassembling OA‐AuNPs with OC‐IONPs into polymer nanobeads. The synthesized MPNAs exhibit a typical core–shell structure, wherein OA‐AuNPs preferentially aggregate and form a plasmonic core and OC‐IONPs assemble a magnetic shell. This unique core–shell heterostructure provides an efficient spatial separation of OA‐AuNPs and OC‐IONPs, which minimizes the interference between them. Meanwhile, the OC‐IONPs distributed in the shell layer achieved the maximum retention of magnetic responsiveness property due to the absence of magnetic shielding from the plasmonic component, which is often encountered in conventional core–shell‐structured MPNSs. Given the rational spatial distribution of magnetic and plasmonic components of OC‐IONPs and OA‐AuNPs, respectively, the self‐assembled MPNAs were characterized with highly retained magnetic properties (≈79.5% of the saturation magnetization of the initial OC‐IONPs) and enhanced plasmonic activities. This excellent magnetic–plasmonic performance endows the resultant MPNAs with great potential for simultaneous magnetic manipulation and optical detection. Using the intrinsic dual functionality of MPNAs as signal transducer and magnetic separator, we demonstrate their potential as bifunctional probes for improving the detection performance on lateral flow immunoassay (LFIA) platform. In summary, our proposed core–shell heterostructure with combined magnetic and plasmonic properties can serve as a novel nanoanalytical platform for the separation and concentration of target compounds from complex biological samples using magnetic properties and simultaneous optical sensing using plasmonic properties.

## 2 Results and Discussion

### 2.1 Synthesis and Characterization of MPNAs

**Scheme 1:**
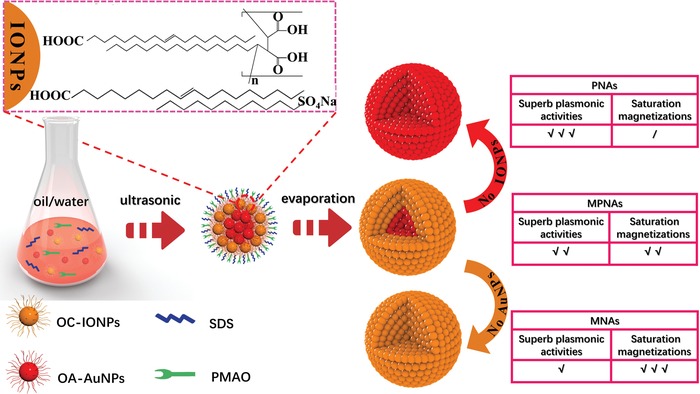
Schematic illustration for the synthetic procedures of MPNAs, PNAs, and MNAs.

**Figure 1:**
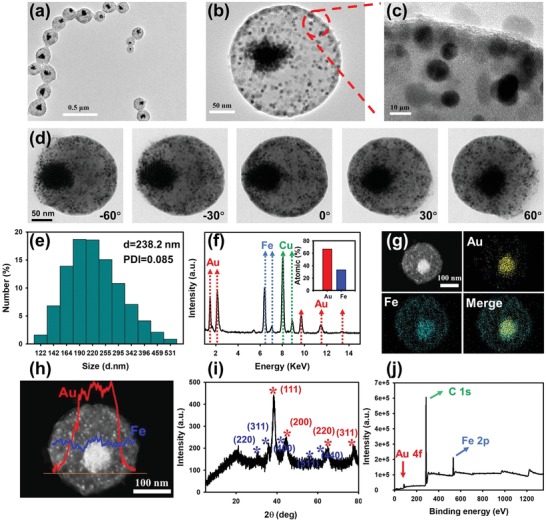
Synthesis and characterizations of the MPNAs. a–c) TEM images of MPNAs at different magnifications. Scale bars, 500 (a), 50 (b), and 10 (c) nm, respectively. d) Tomographic TEM images of MPNA_7:3_ at different tilting angles (left to right: −60°, −30°, −0°, 30°, and 60°). e) Hydrodynamic diameter distribution of the MPNAs in water. f) EDS spectroscopy. g) HAADF‐STEM EDS elemental mapping. h) EDS elemental line scan result. i) Power XRD pattern. j) XPS analysis.

OA‐AuNPs (10 nm, Figure S1a, Supporting Information) and OC‐IONPs (10 nm, Figure S1b, Supporting Information) were used as the building blocks to demonstrate the successful synthesis of multifunctional MPNAs. The schematic of the self‐assembly process for preparing MPNAs is shown in **Scheme** 1. In a typical procedure, OA‐AuNPs, OC‐IONPs, and PMAO were dispersed in chloroform and then transferred to an aqueous solution through micellar encapsulation using sodium dodecyl sulfate (SDS) as a surfactant. After chloroform evaporation, OA‐AuNPs and OC‐IONPs tended to assemble into large and compact nanocomposites. The large‐area transmission electron microscope (TEM) images in **Figure** 1a show that the resulting spherical MPNAs exhibit a characteristic core–shell structure with an average size of 225 nm, which is slightly smaller than the hydrodynamic diameter (*H*_D_) of MPNAs (238 nm) obtained via dynamic light scattering (DLS) measurement (Figure 1e). This slight difference is due to the presence of a PMAO polymer layer on the MPNA surface with a low contrast in TEM imaging. The highly magnified TEM images in Figure 1b demonstrate that an apparent phase separation occurred inside each MPNA nanosphere, wherein OA‐AuNPs preferentially aggregated and formed a plasmonic core, while OC‐IONPs uniformly distributed to assemble into a magnetic shell. The similar self‐assembled behaviors of OA‐AuNPs aggregating and OC‐IONPs distributing evenly were observed in a single‐component self‐assembly system with OA‐AuNPs or OC‐IONPs as the building blocks (Figure S2, Supporting Information). Further high‐resolution TEM images in Figure 1c exhibit a clear distinction between OA‐AuNPs and OC‐IONPs due to their different electron penetrabilities, where OA‐AuNPs demonstrated a higher contrast than OC‐IONPs, thereby causing darker dots in the TEM imaging. Subsequently, an electron tomographic imaging analysis was employed to investigate the core–shell structure of the assembled MPNA. Figure 1d and Movie S1 (Supporting Information) showed the typical tomographic TEM images of our MPNA at different tilting angles ranged from −60° to 60°, where the OC‐IONPs were spatially arranged around the aggregated OA‐AuNPs in 3D space, validating the successful formation of the core–shell‐heterostructured MPNA. In addition, these tomographic TEM images also revealed the off‐center location of the aggregated OA‐AuNPs. To further investigate the morphology and distribution of OA‐AuNPs and OC‐IONPs inside the MPNAs, energy‐dispersive X‐ray spectroscopy (EDS) and elemental mapping, high‐angle annular dark‐field scanning TEM (HAADF‐STEM) elemental mapping, powder X‐ray diffraction (XRD), and X‐ray photoelectron spectroscopy (XPS) were conducted. As shown in Figure 1f, EDS indicates the presence of strong Au and Fe signals in the resultant MPNAs with an elemental composition of 66.8% Au and 33.2% Fe, which is in accordance with the initial feeding amounts of OA‐AuNPs and OC‐IONPs. The HAADF‐STEM and EDS elemental mappings show that Au elements are centered in the core location, whereas Fe elements are uniformly and homogeneously distributed in the outer shell of MPNAs (Figure 1g). The EDS elemental line scan further verifies the core–shell heterostructure of the designed MPNAs (Figure 1h). In addition, the XRD pattern (Figure 1i) and XPS spectrum (Figure 1j and Figure S3 (Supporting Information)) prove the coexistence of OA‐AuNPs and OC‐IONPs in the assembled MPNAs. These results demonstrate the feasibility of the coassembling of OA‐AuNPs and OC‐IONPs into core–shell‐heterostructured MPNAs via emulsion‐based self‐assembly strategy. Moreover, a series of similar core–shell‐structured nanoassemblies with average sizes of 200 nm was obtained using trichloroethylene, methylbenzene, or benzene as the organic phase instead of chloroform (Figure S4, Supporting Information). This finding suggests the versatility of the coassembling method using microemulsion. With these observations, we speculate that the formation of the well‐defined core–shell heterostructure of MPNAs is likely due to the solubility difference of OA‐AuNPs and OC‐IONPs in the PMAO phase and the hydrophobic interactions that can drive the directed self‐assembly of OA‐AuNPs and OC‐IONPs. To better describe the magnetic and plasmonic properties of the proposed MPNAs, two control nanospheres with sizes of ≈200 nm, including plasmonic (PNA_10_, using 10 mg OA‐AuNPs as building blocks) and magnetic nanoassemblies (MNA_10_, using 10 mg OC‐IONPs as building blocks), were synthesized using the same self‐assembly protocols and further confirmed by TEM and DLS analysis (Figure S2, Supporting Information).

### 2.2 Highly Retained Magnetic and Plasmonic Activities of MPNAs

**Figure 2:**
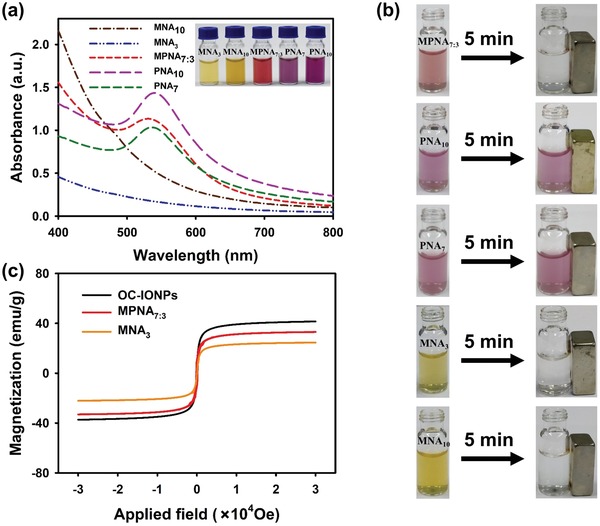
Characterization of magnetic and optical performances of the MPNA_7:3_. a) UV–vis absorption spectra of MNA_10_, MNA_3_, MPNA_7:3_, PNA_7_, and PNA_10_ at the same particle concentrations (15 × 10^−12^ m). b) Photographs of MNA_10_, MNA_3_, MPNA_7:3_, PNA_7_, and PNA_10_ collected by an external magnetic field. c) Magnetic hysteresis loops of OC‐IONPs, MNA_3_, and MPNA_7:3_ obtained on a SQUID system at 300 K.

The magnetic and plasmonic activities of MPNAs are mainly determined by the inherent geometric structure of nanocomposites and the relative mass ratio of magnetic and plasmonic components in individual nanocomposite. The assembled MPNAs reveal a typical core–shell nanostructure, in which the magnetic and plasmonic components were separated spatially, thereby minimizing the interference with each other. Meanwhile, OA‐AuNPs distributed inside the MPNAs as a core, thereby eliminating the shielding effect of Au shell on the magnetic response and consequently obtaining high saturation magnetization. To better balance the magnetic and plasmonic activities of MPNAs, the relative mass ratio of magnetic and plasmonic components inside individual MPNAs was regulated by altering the feeding ratio of OA‐AuNPs and OC‐IONPs during the self‐assembly. The synthesis conditions and characterization results of the MPNAs are summarized in Table S1 (Supporting Information). Results show that the MPNAs that were synthesized at various feeding ratios of OA‐AuNPs and OC‐IONPs from 5:5 to 9:1 have approximately equal particle diameters (200–250 nm) with polydispersity indices of ≈0.1, indicating high synthesis stability and reproducibility. The UV–vis absorption spectra present a slight redshift in the maximum absorption peak from 522 to 538 nm when the feeding ratio of OA‐AuNPs and OC‐IONPs was increased from 5:5 to 9:1. As a result, the prepared MPNAs demonstrate color changes from orange, to red, and to amaranth as the feeding ratios of OA‐AuNPs and OC‐IONPs increased from 5:5 to 9:1 (**Figure** 2a and Figure S5 (Supporting Information)). Moreover, Table S1 (Supporting Information) indicates that the maximum absorbance increased from 0.69 to 1.35 with the increase in the feeding ratio, which is attributed to the increasing OA‐AuNP doping amount inside a single MPNA. By contrast, the saturation magnetization gradually decreased from 54.8 to 10.3 emu g^−1^ following the reduction of OC‐IONPs' doping amount. The magnetic recoveries, defined as the percentage of the MPNA content before and after magnetic separation under an applied magnetic field, were greatly reduced from 98.5% to 74.9%. Therefore, to ensure the enhanced plasmonic activities and maintain the high saturation magnetization, the optimized feeding ratio of OA‐AuNPs and OC‐IONPs at 7:3 was selected and applied for the self‐assembly synthesis of MPNAs (marked as MPNA_7:3_). The magnetic and optical properties of five different nanoassemblies, including MNA_10_, MNA_3_ (using 3 mg OC‐IONPs as building blocks), MPNA_7:3_, PNA_7_ (using 7 mg OA‐AuNPs as building blocks), and PNA_10_, are illustrated in Figure 2a,b. The magnetic properties of the MNA and MPNA nanostructures were immediately observed after the synthesis and utilization (washing and collecting) of these nanocomposites. Figure 2b shows that the MNA_10_, MNA_3_, and MPNA_7:3_ nanocomposites were readily collected by an external magnetic field. However, no evident magnetic responsiveness was observed for the PNAs (PNA_7_ and PNA_10_). The inset of Figure 2a illustrates that the color of the MNA solution is light yellow, whereas the solution color of MPNA_7:3_ and PNAs (PNA_7_ and PNA_10_) is wine red and amaranth, respectively. The absorption spectra of MNAs, MPNA_7:3_, and PNAs are presented in Figure 2a, in which the MPNA_7:3_ nanocomposite shows remarkably higher absorbance than the conventional MNAs (MNA_10_ and MNA_3_) at the same particle concentrations (15 × 10^−12^ m). The enhanced absorbance of MPNA_7:3_ benefited from the collective light absorption of numerous assembled small‐sized OA‐AuNPs, which contributes to increasing the analytical sensitivity in the absorption‐based detection methods. Moreover, we also found that the absorbance of MPNA_7:3_ was lower than that of PNA_10_ because the presence of magnetic component of OC‐IONPs inside the MPNAs decreases the relative mass ratio of the plasmonic component. However, the absorbance of the MPNA_7:3_ was about 1.1‐fold higher than that of PNA_7_ because the assembled OC‐IONPs in MPNA_7:3_ provided synergistically enhanced absorbance relative to the pure PNA_7_. Unlike PNAs, MPNA_7:3_ exhibits excellent magnetic responsiveness, indicating its potential for magnetic‐related applications. Figure 2c shows that the magnetization curve of MPNAs exhibits super‐paramagnetism under a saturation magnetization of 33.1 emu g^−1^. The saturation magnetization of MPNAs reached up to 79.5% of pure OC‐IONPs, which is about 1.34‐fold higher than that of the MNA_3_ (24.7 emu g^−1^, ≈59.3% of that of pure OC‐IONPs). The possible reason is that OA‐AuNPs preferentially aggregate and form a plasmonic core, occupying the inner space of MPNA_7:3_ to result in the OC‐IONPs to mainly concentrated in the outer layer; by contrast, for MNA_3_, the OC‐IONPs evenly distributed throughout the interior of the nanoparticle, thus causing smaller saturation magnetization relative to our MPNA_7:3_. On the other hand, the OA‐AuNPs aggregated and distributed in the core location, which minimized the magnetic shielding that originated from the plasmonic component, thereby preserving the high saturation magnetization of the MPNAs.

**Figure 3:**
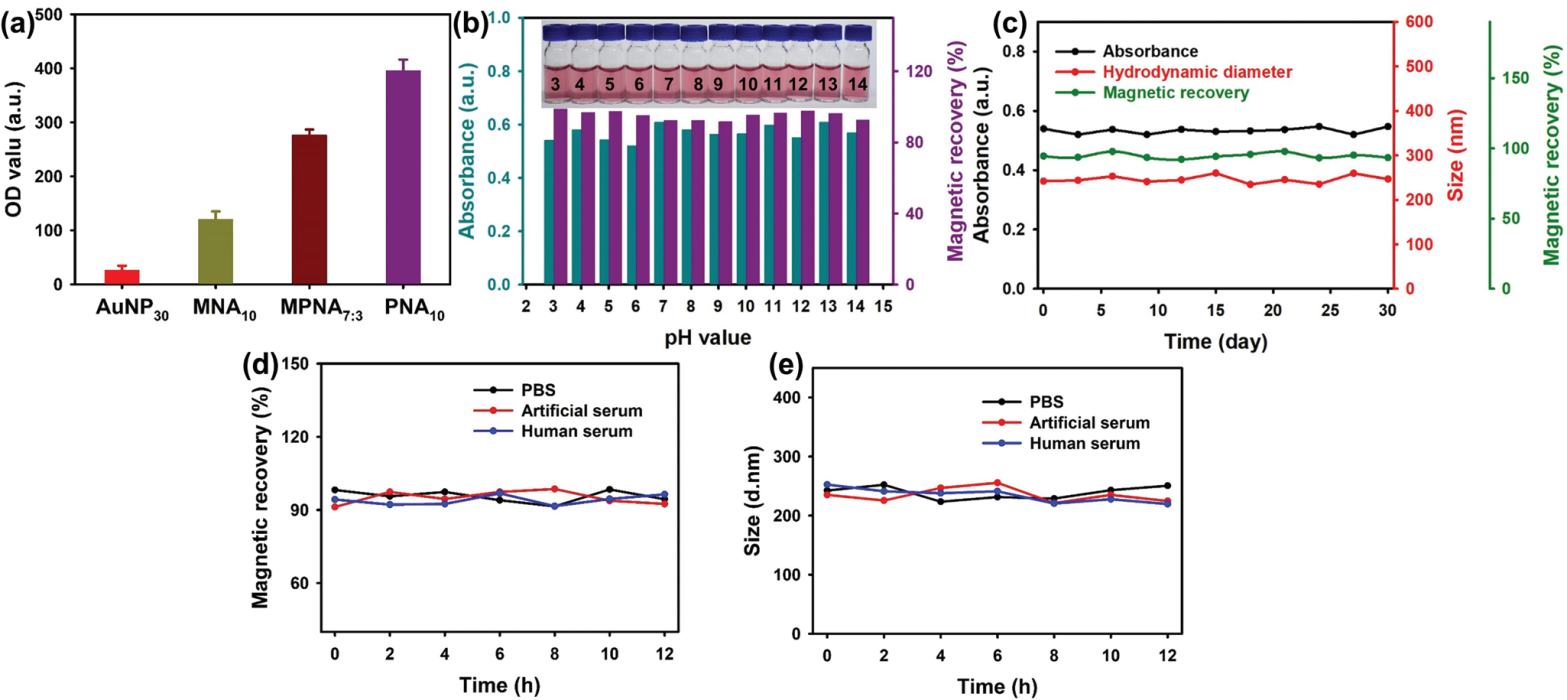
a) The corresponding OD values of AuNP_30_, MNA_10_, MPNA_7:3_, and PNA_10_ at the same molar concentration of 30 × 10^−12^ m after being sprayed onto the NC membrane as T line. b) Absorbance and magnetic recovery of the MPNA_7:3_ water solutions under different pH values. Inset exhibits the photographs of the MPNA_7:3_ dispersions with various pH values. c) Hydrodynamic diameter, absorbance, and magnetic recovery of the MPNA_7:3_ against long‐term storage for 30 days. d) Magnetic recovery and e) hydrodynamic diameter of the MPNA_7:3_ dispersed in PBS, artificial serum, and human serum.

To further explore the effect of the obtained MPNA_7:3_ on the optical density (OD) value at the T line, four different nanostructures, including 30 nm AuNPs (AuNP_30_), MNAs, MPNA_7:3_, and PNAs at the same molar concentration of 30 × 10^−12^ m, were sprayed onto the nitrocellulose (NC) membrane as the T line. The corresponding OD values were then collected using a commercial HG‐8 strip reader. The results show that the OD value of the MPNA_7:3_ reached up to 276.5, which was ≈10.7‐fold and 2.3‐fold higher than those of conventional AuNP_30_ and MNA_10_ (**Figure** 3a). The high OD value of the MPNA_7:3_ nanocomposite on the strip was mainly attributed to the collective absorbance of the whole assembled small‐sized AuNPs. Furthermore, PNAs revealed the highest OD values at the T line (≈1.4‐fold higher than that of MPNA_7:3_), which is mainly due to the smaller mass percent of AuNPs in MPNA_7:3_ (70%) relative to pure PNAs (100%). These findings indicate that the assembled MPNA_7:3_ and PNAs can serve as amplified colorimetric labels for the further improvement of the detection sensitivity of absorbance‐dominated signal output strategies. As revealed in Figure 3b, further UV–vis absorption spectrum and magnetic recovery analyses of MPNA_7:3_ showed that the absorbance at 530 nm and the magnetic recovery efficiency exhibit negligible changes against pH variations from 3 to 14. This finding indicates the high colloidal, optical, and magnetic stabilities of MPNA_7:3_ in solution within a wide pH range. Figure 3c illustrates that no evident changes were observed in the absorbance at 530 nm, the magnetic recovery efficiency, and the *H*_D_ of MPNA_7:3_ after storage for 30 days. This finding indicates the excellent long‐term storage stability and absence of leakage or degradation of MPNA_7:3_ during storage. Figure 3d,e showcases that the MPNA_7:3_ dispersed in buffer, artificial serum, and human serum solutions still maintained their original *H*_D_ and magnetic recovery efficiency after 12 h of observation, implying the high colloidal stability of MPNAs for potential biological applications. The excellent colloidal, optical, and magnetic stabilities can be attributed to the well‐retained oleylamine or oleic acid ligand passivation and the spatial isolation of OA‐AuNPs and OC‐IONPs from the surrounding environments due to the polymer surface layers coated onto MPNAs.

### 2.3 Highly Sensitive Quantification of Hepatitis C Virus Antibody (HCV‐Ab) in Serum via MPNA–LFIA Strips

**Scheme 2:**
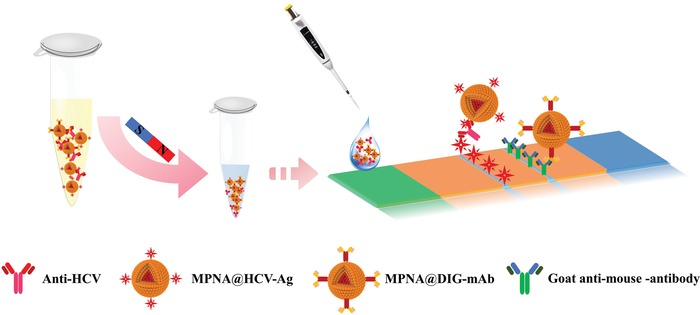
Schematic representation for anti‐HCV detection using the double antigen sandwich MPNA_7:3_–LFIA strip platform.

**Figure 4:**
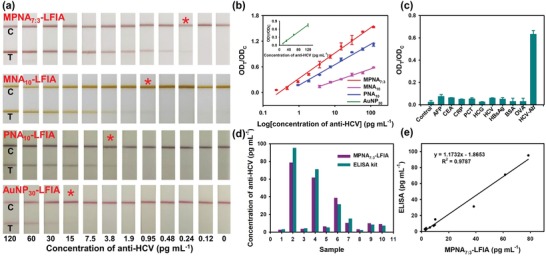
a) The reacted strip prototype photographs of all four LFIA strips, including MPNA_7:3_–LFIA, MNA_10_–LFIA, PNA_10_–LFIA, and AuNP_30_–LFIA under various anti‐HCV concentrations ranged from 0 to 120 pg mL^−1^. b) The linear relationship for anti‐HCV detection based on these four strips within the concentration range of 0–120 pg mL^−1^. c) The specificity evaluation of MPNA_7:3_–LFIA for anti‐HCV by measuring the signal response against other common interfering proteins. d,e) The correlation analysis of the detected concentrations of anti‐HCV between the MPNA_7:3_–LFIA method and a commercial ELISA kit in detecting ten HCV‐positive human serum samples.

Encouraged by the highly retained magnetic and plasmonic activities of MPNAs, the feasibility of using MPNAs as novel difunctional probes was investigated on the LFIA test strip platform, which is a powerful point of care (POC) diagnostic tool.^19^ In this case, the MPNA_7:3_ nanocomposites were applied for the fabrication of MPNA–LFIA strip due to its balanced magnetic and plasmonic activities. HCV infection causes various liver diseases, such as cirrhosis, hepatocellular carcinoma, and liver failure with substantial morbidity and mortality.^20^ Several serological testing methods, including enzyme‐linked immunosorbent assay (ELISA)^21^ and nucleic acid testing,^22^ are used to diagnose HCV infections. Among them, the serologic detection of antibody against HCV (anti‐HCV) using ELISA is considered as the first‐line diagnosis in clinics.^23^ However, the routine POC diagnostic utilization of this method is hampered by the complicated operation and long detection time. Thus, a sensitive, rapid, and accurate determination of anti‐HCV is highly anticipated for monitoring the progression and prognosis of liver diseases. For conjugation with recognition molecules such as antigens, MPNA_7:3_ NPs were dispersed in alkaline water to hydrolyze the anhydride of PMAO into carboxyl groups. The carboxylated MPNA_7:3_ nanosphere was then modified with hepatitis C virus antigen (HCV‐Ag) via 1‐ethyl‐3‐(3‐dimethylaminopropyl) carbodiimide (EDC)‐mediated covalent coupling to form MPNA_7:3_@HCV‐Ag as detection probes, which increased *H*_D_ from 238.2 to 252.4 nm (Figure S6, Supporting Information). Using the same method, anti‐digoxin monoclonal antibody (anti‐DIG mAb) was modified onto the MPNA_7:3_ surface to prepare the MPNA_7:3_@anti‐DIG mAb as control probes, which is independent of the anti‐HCV concentration in the sample solution and used as a reference signal on the C line to enable a reliable ratiometric detection using the OD_T_/OD_C_ value (the ratios of signals on the T line to those of the C line) as quantitative signals.^24^ The principle of the designed MPNA_7:3_–LFIA strip for the sensitive detection of anti‐HCV in human serum is presented in **Scheme** 2. MPNA_7:3_@HCV‐Ag and MPNA@anti‐DIG mAb were first added and mixed with 350 µL of human serum solution to capture anti‐HCV and form the MPNA_7:3_@HCV‐Ag–anti‐HCV complex. The complex was then collected magnetically and resuspended in 70 µL of phosphate buffer solution (PBS) to run the LFIA test strip that was preprepared by dispensing HCV antigen and goat anti‐mouse IgG on the NC membrane as the respective T and C lines. For direct comparison, we benchmarked the performance of MPNA_7:3_ in LFIA against MNA_10_, PNA_10_, and AuNP_30_ with the same set of antibodies and materials on the double antigen sandwich LFIA strip design. To obtain the highest sensitivity and appropriate OD values at the T and C lines, the MPNA_7:3_–LFIA strip development and optimization involved the MPNA_7:3_ modification with HCV antigen (including the pH value in Figure S7a (Supporting Information), the EDC concentration in Figure S7b (Supporting Information), and the labeling amount of HCV antigen in Figure S7c (Supporting Information)), the concentration of HCV antigen sprayed on the T line (Figure S7d, Supporting Information), the used dosage of MPNA_7:3_@HCV‐Ag probes in each strip (Figure S7e, Supporting Information), and the immunoreaction time for signal interpretation (Figure S7f, Supporting Information). The optimized conditions that can maximize the OD value at the T line are shown in Figure S7 (Supporting Information). Under the developed conditions, a series of human serum sample solutions containing different concentrations of anti‐HCV within the range of 0–120 pg mL^−1^ was measured via MPNA_7:3_–LFIA, MNA_10_–LFIA, PNA_10_–LFIA, and AuNP_30_–LFIA. Then, a systematical comparison in the detection performance of all four LFIA strips was conducted on the basis of qualitative and quantitative assays using visual inspection and a commercial HG‐8 strip reader, respectively. For anti‐HCV qualitative assay, the visual limit of detection (vLOD), which is defined as the lowest anti‐HCV concentration required for producing a visible red band at the T line, was determined. The reacted strip prototype photographs under different target anti‐HCV concentrations are summarized in **Figure** 4a. The results indicated that the vLOD of the proposed MPNA_7:3_–LFIA was 0.24 pg mL^−1^, which is ≈4.0‐fold, 15.8‐fold, and 62.5‐fold lower than those of MNA–LFIA, PNA–LFIA, and AuNP_30_–LFIA, respectively. For anti‐HCV quantitation, the detection results for anti‐HCV were described by plotting the variations of OD_T_/OD_C_ values against the concentrations of target anti‐HCV. As shown in Figure 4b, excellent linear relationships between the OD_T_/OD_C_ values and the anti‐HCV concentrations were observed in all four LFIA strips. The MPNA_7:3_–LFIA strip exhibited the widest linear detection range (0.24–120 pg mL^−1^) and the lowest LOD value of 0.24 pg mL^−1^ (calculated by the blank plus threefold standard deviation). Compared with the conventional AuNP_30_–LFIA strip, the sensitivity of the proposed MPNA_7:3_–LFIA strip displayed an ≈65.3‐fold improvement, which is mainly due to the significantly enhanced plasmonic activities of the MPNA_7:3_ and the cleanup and concentration of the target through the additional magnetic separation step. Moreover, compared with the magnetic separation‐assisted MNA–LFIA strip, the MPNA_7:3_–LFIA method demonstrated a fourfold increase in the sensitivity because of the higher absorbance of MPNA_7:3_ than MNAs. Although the assembled PNAs possessed stronger optical absorption than MPNA_7:3_, the sensitivity of the PNA–LFIA strip was ≈14.7‐fold lower than that of our MPNA_7:3_–LFIA approach. The possible reason for this phenomenon is that MPNA_7:3_ revealed an excellent magnetic responsiveness for the magnetic manipulation to separate and concentrate the trace target analytes from complex biological samples and eliminate the influence of matrix interference on the MPNA_7:3_–LFIA strip. These results demonstrated that the designed MPNA_7:3_ nanospheres with highly retained plasmonic and magnetic activities are well suitable as amplified dual functional probes for improving the LFIA sensitivity. To investigate the specificity of the MPNA_7:3_–LFIA strip against anti‐HCV recognition, several common interfering proteins, including alpha fetoprotein, carcinoembryonic antigen, C‐reaction protein, procalcitonin, human chorionic gonadotropin, HCV Ag, hepatitis B surface antigen, bovine serum albumin (BSA), and ovalbumin were selected as reference substances. As shown in Figure 4c, a high signal response was observed in measuring anti‐HCV (7.5 pg mL^−1^), whereas negligible signal responses were obtained in testing other interfering proteins (1 µg mL^−1^), suggesting the excellent selectivity of the proposed MPNA_7:3_–LFIA strip for anti‐HCV detection. Accuracy and precision estimation of the proposed MPNA_7:3_–LFIA was completed by determining the intra‐ and inter‐assay recoveries and coefficients of variation (CV) of five anti‐HCV‐spiked serum samples with target concentrations of 3.8, 7.5, 15, 30, and 60 pg mL^−1^. As shown in Table S2 (Supporting Information), the average recoveries for intra‐ and inter‐assay varied from 90.43% to 111.34%, and the CV was less than 11%, suggesting an acceptable accuracy and precision for anti‐HCV quantitation. Given its high sensitivity, excellent selectivity, and good precision, the MPNA_7:3_–LFIA strip was further extended for clinical monitoring of HCV infection in real serum. ELISA was employed to evaluate the detection accuracy and reliability of the MPNA_7:3_–LFIA strip. Ten HCV‐positive serum samples with different anti‐HCV concentrations were simultaneously detected using the MPNA_7:3_–LFIA strip and a commercial ELISA kit. The results obtained by the MPNA_7:3_–LFIA agreed well with those of ELISA (Figure 4d and Table S3 (Supporting Information)), and a high linear correlation (*R* ^2^ = 0.9787, Figure 4e) was obtained between the two methods, showing the high accuracy of the MPNA_7:3_–LFIA in the quantitative determination of anti‐HCV. Time‐resolved fluoro‐immunoassay (TRIFMA) is the most popular routine screening technology to determine anti‐HCV in real clinical serum samples. We further compared the analytical performance of MPNA_7:3_–LFIA with that of a commercial TRIFMA kit. Thirty‐two HCV‐positive serum samples from hepatitis C patients and 20 HCV‐negative serum samples from healthy volunteers were simultaneously detected by the two methods. As displayed in Table S4 (Supporting Information), all HCV‐negative samples were tested negative using MPNA_7:3_–LFIA and TRIFMA. By contrast, all HCV‐positive samples were correctly detected (i.e., positive) using the proposed MPNA_7:3_–LFIA, whereas eight out of 32 positive samples were determined to be negative using TRIFMA. The possible reason is that the anti‐HCV concentrations in the eight serum samples are below the LOD value of TRIFMA. In conclusion, the proposed MPNA_7:3_–LFIA strip possesses a higher accuracy than TRIFMA in the rapid diagnostic testing of anti‐HCV in real serum samples, indicating that the proposed method can serve as an alternative for TRIFMA in the clinical routine screening of HCV infections because of its simplicity, higher sensitivity, shorter analysis time, and better compatibility for routine POC diagnostic applications.

## 3 Conclusions

Herein, we report the successful synthesis of colloidal core–shell‐heterostructured MPNAs by coassembling OC‐IONPs and OA‐AuNPs into polymer nanobeads. Owing to the spatial separation of plasmonic core and magnetic shell, the current MPNAs could efficiently prevent the reduction in the saturation magnetization of the magnetic components caused by the intrinsic magnetic shielding of the plasmonic components compared with that of conventional core–shell gold‐coated magnetic NPs. Moreover, the plasmonic activity of MPNAs was significantly enhanced by regulating the feeding ratio of OC‐IONPs and OA‐AuNPs to achieve the maximized loading of OA‐AuNPs. Taking advantages of the highly retained saturation magnetization and enhanced plasmonic signal transducer, the assembled MPNAs were integrated with LFIA strip platform for the simultaneous magnetic manipulation and ultrasensitive optical detection of anti‐HCV in human serum with a LOD of 0.24 pg mL^−1^, providing at least fourfold improvement in sensitivity compared with three other control groups of MNA_10_, PNA_10_, and AuNP_30_. Collectively, our designed core–shell‐heterostructured MPNAs are a promising magnetic–plasmonic nanoanalysis platform for the separation and concentration of target analytes from complex biological samples using magnetic properties and simultaneous optical sensing using plasmonic properties. Considering the versatility of our developed self‐assembly strategy, we believe that the assembled composite multifunctional NPs with various components can be readily realized using the proposed approach.

## 4 Experimental Section

*Materials*: HAuCl_4_·3H_2_O, FeCl_2_·4H_2_O, FeCl_3_, oleylamine, oleic acid, BSA, SDS, EDC, trisodium citrate, and PMAO (molecular weight, MW = 30 000–50 000 Da) were purchased from Sigma‐Aldrich. The sample and absorbent pads and nitrocellulose membrane were purchased from Wuxi Zodolabs Biotech Co., Ltd. Goat anti‐mouse IgG and HCV‐Ag were obtained from Chongqing Xinyuanjiahe Biotechnology Inc. Human serum containing HCV‐Ab was collected from Jiangxi Provincial People's Hospital (Nanchang, China). Mouse anti‐DIG mAb was obtained from Abcam. The ELISA kit was purchased from Huding Biotechnology (Shanghai, China). All chemicals were of analytical grade and purchased from Sinopharm Chemical Corp. without further purification unless specified. Millipore water was obtained from a Milli‐Q purification system.

*Instrument and Characterization*: The morphology and structure of the resultant MPNAs were observed using a high‐resolution TEM (JEOL JEM 2100, Tokyo, Japan). The hydrodynamic size distribution of MPNAs was measured using a scientific DLS NP analyzer (Malvern Nano ZSE, London, UK). The UV–vis absorption spectra were obtained using a Genesys 10s UV–vis spectrophotometer (Thermo Scientific, Waltham, MA). Fluorescence spectra were collected via a Hitachi F‐7000 fluorescence spectrophotometer. XRD analysis was performed on a D8 ADVANCE X‐Ray powder diffractometer (Bruker, Germany). XPS was conducted on a Thermo Scientific K‐Alpha system with a 75 eV pass energy and 0.05 eV binding energy steps. The magnetic properties of the prepared MPNAs were characterized on a superconducting quantum interference device at 300 K.

*Synthesis of OA‐AuNPs*: OA‐AuNPs (10 nm) were synthesized according to a previously reported method.^25^ First, 100 mg of HAuCl_4_·3H_2_O was dissolved in a mixed solution containing 1.0 mL of toluene and 1.2 mL of oleylamine. Then, the mixture was added to a solution containing 20 mL of toluene and 20 mL of oleylamine, which was previously heated to 110 °C for 6 h. After cooling the mixed solution to room temperature, an equal volume of absolute ethanol was added for overnight reaction. Afterward, the precipitate was resuspended in 3.0 mL of chloroform and stored at 4 °C for further use.

*Synthesis of OC‐IONPs*: OC‐IONPs (10 nm) were synthesized according to a previously reported method.^26^ Briefly, 1.59 g of FeCl_2_·4H_2_O and 2.59 g of FeCl_3_ were fully dissolved in 150 mL aqueous solution, and then the mixture was heated to 50 °C and bubbled with N_2_ for 15 min. Subsequently, 12.5 mL of NH_3_·H_2_O (25% v/v) was quickly added into the mixture under vigorous stirring at 500 rpm for 30 min. The solution color changed from yellow to black. Afterward, the formed precipitate was collected and washed for 5 times with neutral pH water under an additional magnetic field. The precipitates were ultrasonically dissolved in 100 mL of water. After adding 1.2 mL of OC, the dark suspension was stirred for 3 h at 70 °C under N_2_ protection. Finally, the synthetic oily OC‐IONP product was washed with ethanol and then resuspended in chloroform for further use.

*Synthesis of MPNA_7:3_*: The MPNAs were prepared by coassembling OA‐AuNPs and OC‐IONPs into a polymer matrix via an evaporation‐mediated emulsification technique. First, 5 mg of PMAO, 7 mg of OA‐AuNPs, and 3 mg of OC‐IONPs were completely dissolved in 100 µL of chloroform to form an oil phase. Then, 250 µL of water solution containing 1.25 mg of SDS, which was used as the aqueous phase, was added into the oil phase. The mixture was emulsified via ultrasound using an ultrasonicator for 2 min (working, 5 s; pausing, 10 s) at a power of 76.8 W. The resultant solution was then placed in the oven at 60 °C for 4 h. With the chloroform evaporation, the OA‐AuNPs and OC‐IONPs were assembled into large‐sized MPNA_7:3_. The formed MPNA_7:3_ was then washed with ultrapure water via centrifugation at 13 500 rpm for 15 min, and the particulates were resuspended in alkaline water (pH 10) for 24 h to hydrolyze the anhydride of PMAO and produce numerous carboxyl groups for subsequent surface functionalization. The carboxylated MPNA_7:3_ was obtained via centrifugation at 13 500 rpm for 15 min and dissolved in ultrapure water for further use.

*Synthesis of MPNA_7:3_@HCV‐Ag*: The MPNA_7:3_@HCV‐Ag conjugates were synthesized via the covalent coupling of MPNA_7:3_ and HCV‐Ag. In brief, 10 µL of the as‐prepared MPNA_7:3_ (12 mg mL^−1^), 5 µg of HCV‐Ag, and 10 µg of EDC were dissolved in 0.01 m pH 7.4 PBS, and the mixture was incubated at room temperature for 30 min under gentle stirring. Then, 100 µL of BSA (10% w/v) and 10 µg of EDC were added into the mixture to allow reaction for 1 h. Finally, the mixture was centrifuged at 13 500 rpm for 15 min, and the precipitates were resuspended in 60 µL of 0.01 m PBS (pH 7.4) containing 25% w/v saccharose, 1% w/v BSA, and 0.1% w/v sodium azide (NaN_3_). The conjugation of MPNA_7:3_ and anti‐DIG mAb (MPNA_7:3_@DIG‐mAb) was conducted using the same synthesis procedure as MPNA_7:3_@HCV‐Ag.

*Fabrication of MPNA_7:3_–LFIA Strip*: The MPNA_7:3_–LFIA strip was constructed according to the previous report.[qv: 19a] First, HCV‐Ag (2 mg mL^−1^) and goat anti‐mouse IgG (1 mg mL^−1^) were sprayed on the NC membrane as the T and C lines, respectively. Then, the modified NC membrane was stored at 37 °C for drying overnight. The sample pads were treated with 20 mmol L^−1^ sodium borate buffer (pH 8.0) containing 1.0% w/v BSA, 0.1% w/v NaN_3_, and 0.25% v/v Tween‐20. The treated NC membrane and sample pads were assembled into test strips together with the absorbent pads, and then packed in a matching plastic case for storage in a dry environment.

*Detection of Anti‐HCV in Serum Using MPNA_7:3_–LFIA Strip*: First, 4 µL of MPNA_7:3_@HCV‐Ag and 1 µL of MPNA_7:3_@DIG‐mAb were added into 350 µL of sample solution to react for 5 min. Then, the formed MPNA_7:3_@HCV‐Ag–anti‐HCV complex and the free MPNA_7:3_@DIG‐mAb were collected using an external magnetic field for 10 min and resuspended in 70 µL of 0.01 m PBS (pH 7.4). Subsequently, the resuspension solution was pipetted into the sample well of the strip. After 15 min, the photographs of the reacted strips and the corresponding ODs at the T and C lines were recorded using a Sony DSC‐HX300 digital camera (Sony, Tokyo, Japan) and a commercial HG‐8 strip reader (Shanghai Huguo Science Instrument Co., Ltd.), respectively.

## Conflict of Interest

The authors declare no conflict of interest.

## Supporting information

Supporting Information

Supporting Information

Supporting Information
