# Supplementary material for: Core–Shell‐Heterostructured Magnetic–Plasmonic Nanoassemblies with Highly Retained Magnetic–Plasmonic Activities for Ultrasensitive Bioanalysis in Complex Matrix
Source: Adv Sci (Weinh). 2019 Dec 13;7(2):1902433. doi: 10.1002/advs.201902433 (PMC6974949; doi:10.1002/advs.201902433)
Supplement: Supplementary file 1 — Supporting Information [file ADVS-7-1902433-s001.pdf]

Copyright WILEY-VCH Verlag GmbH & Co. KGaA, 69469 Weinheim, Germany, 2019.

ADVANCED SCIENCE
Open Access

Supporting Information

for *Adv. Sci.,* DOI: 10.1002/advs.201902433

Core–Shell-Heterostructured Magnetic–Plasmonic Nanoassemblies with Highly Retained Magnetic–Plasmonic Activities for Ultrasensitive Bioanalysis in Complex Matrix

Liangwen Hao, Yuankui Leng, Lifeng Zeng, Xirui Chen, Jing Chen, Hong Duan, Xiaolin Huang, Yonghua Xiong,* and Xiaoyuan Chen*

Copyright WILEY-VCH Verlag GmbH & Co. KGaA, 69469 Weinheim, Germany, 2019.

Supporting Information

Core–Shell Heterostructured Magnetic–Plasmonic Nanoassemblies with Highly Retained Magnetic–Plasmonic Activities for Ultrasensitive Bioanalysis in Complex Matrix

Liangwen Hao, Yuankui Leng, Lifeng Zeng, Xirui Chen, Jing Chen, Hong Duan, Xiaolin Huang, Yonghua Xiong*, and Xiaoyuan Chen*

L. Hao, Dr. Y. Leng, X. Chen, J. Chen, H. Duan, Dr. X. Huang, Prof. Y. Xiong
State Key Laboratory of Food Science and Technology, School of Food Science and Technology, Nanchang University, Nanchang 330047, P. R. China;
E-mail: hxl19880503@163.com; yhxiongchen@163.com

Prof. X. Chen
Laboratory of Molecular Imaging and Nanomedicine (LOMIN), National Institute of Biomedical Imaging and Bioengineering (NIBIB), National Institutes of Health (NIH), Bethesda, Maryland 20892, United States

Dr. L. Zeng
The People's Hospital in Jiangxi Province, Nanchang 330006, PR China

Prof. Y. Xiong
Jiangxi Key Laboratory for Microscale Interdisciplinary Study, Nanchang University, Nanchang 330047, P. R. China

List of content

Figure S1 3

Figure S2 4

Figure S3 5

Figure S4 6

Figure S5 7

Figure S6 8

Figure S7 9

Figure S8 10

Table S1 11

Table S2 12

Table S3 13

Table S4 14

Table S5 15

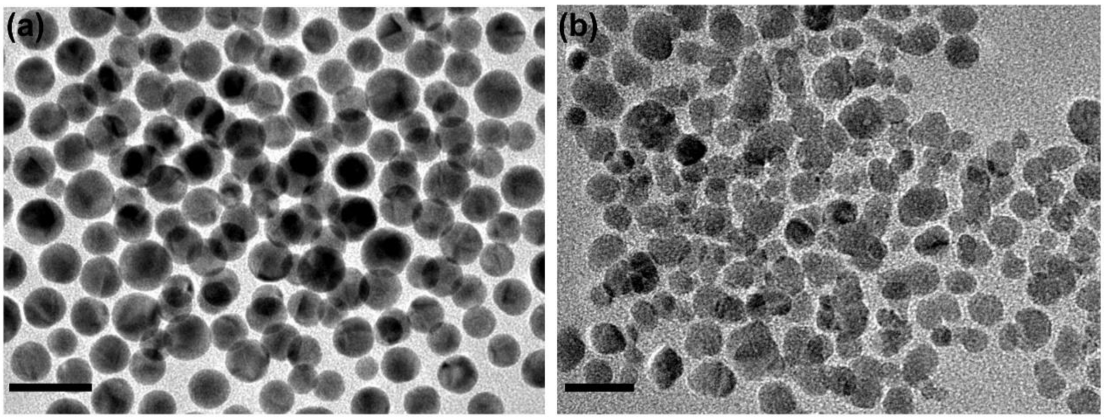

Figure S1. TEM images of OA-AuNPs a) and OC-IONPs b). Scale bar, 10 nm.

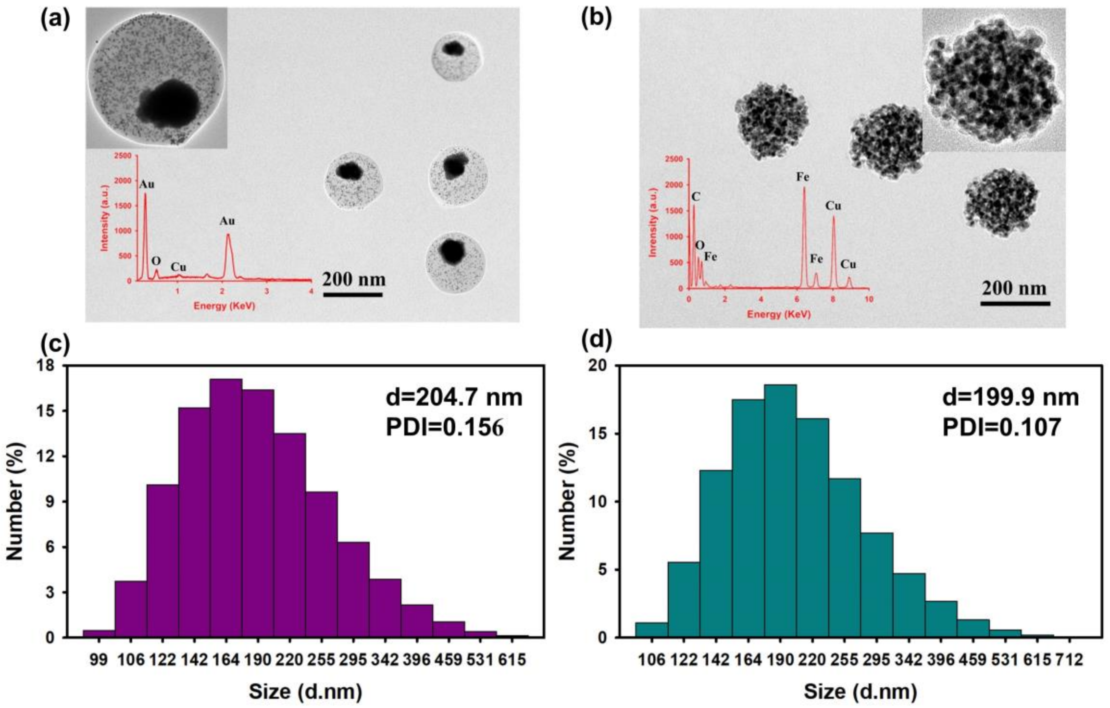

Figure S2. a-b) TEM images of PNAs a) and MNAs b). c-d) Hydrodynamic diameter distribution of PNAs c) and MNAs d) in water. The inset of (a) and (b) are the EDS of PNAs and MNAs, respectively.

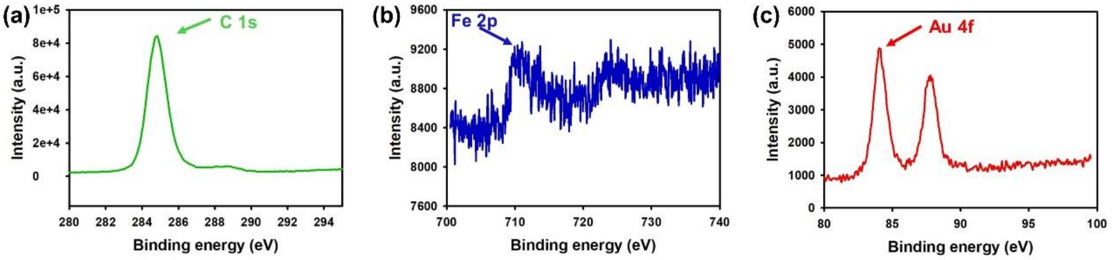

Figure S3. XPS spectrum analysis of MPNAs. High-resolution XPS spectra of C1s a), Fe 2p b) and Au 4f c).

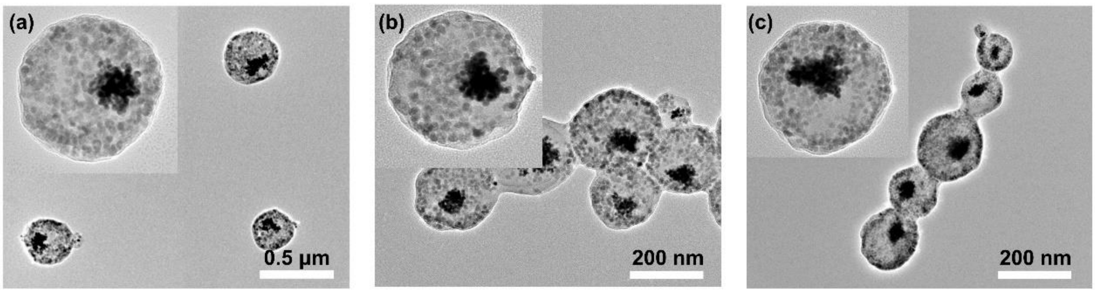

Figure S4. TEM images of MPNAs synthesized using trichloroethylene a), methylbenzene b) and benzene c) as organic phase.

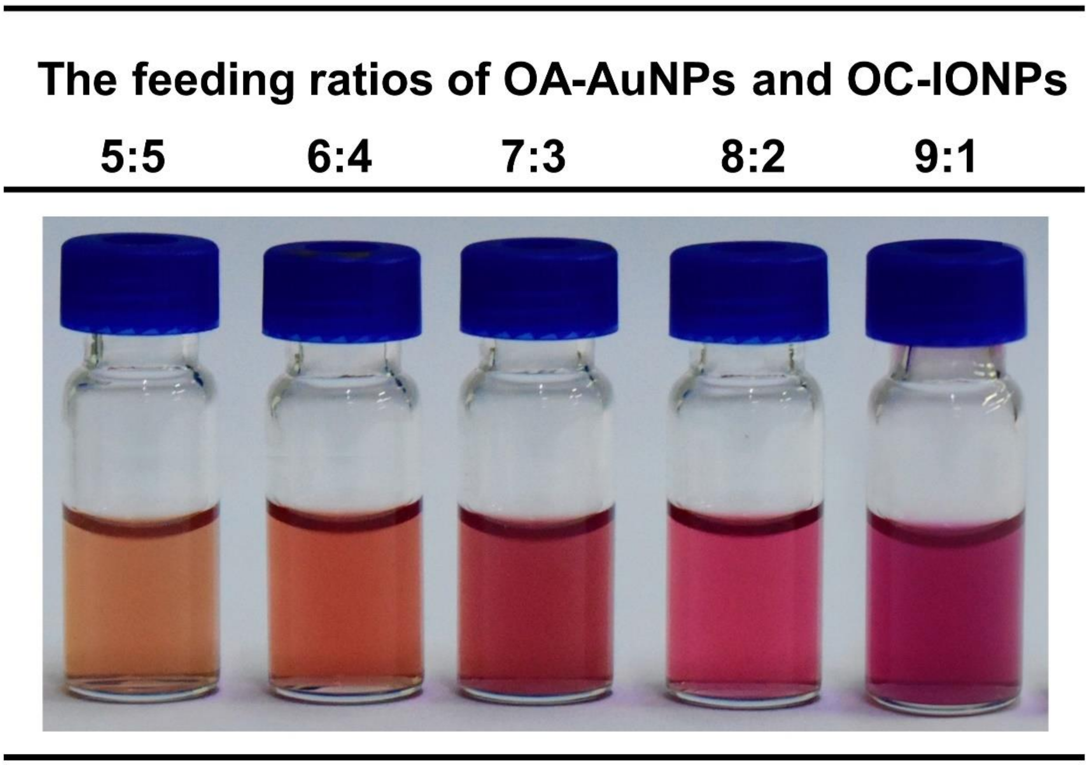

Figure S5. The photograph of MPNAs at different feeding ratios of OA-AuNPs and OC-IONPs

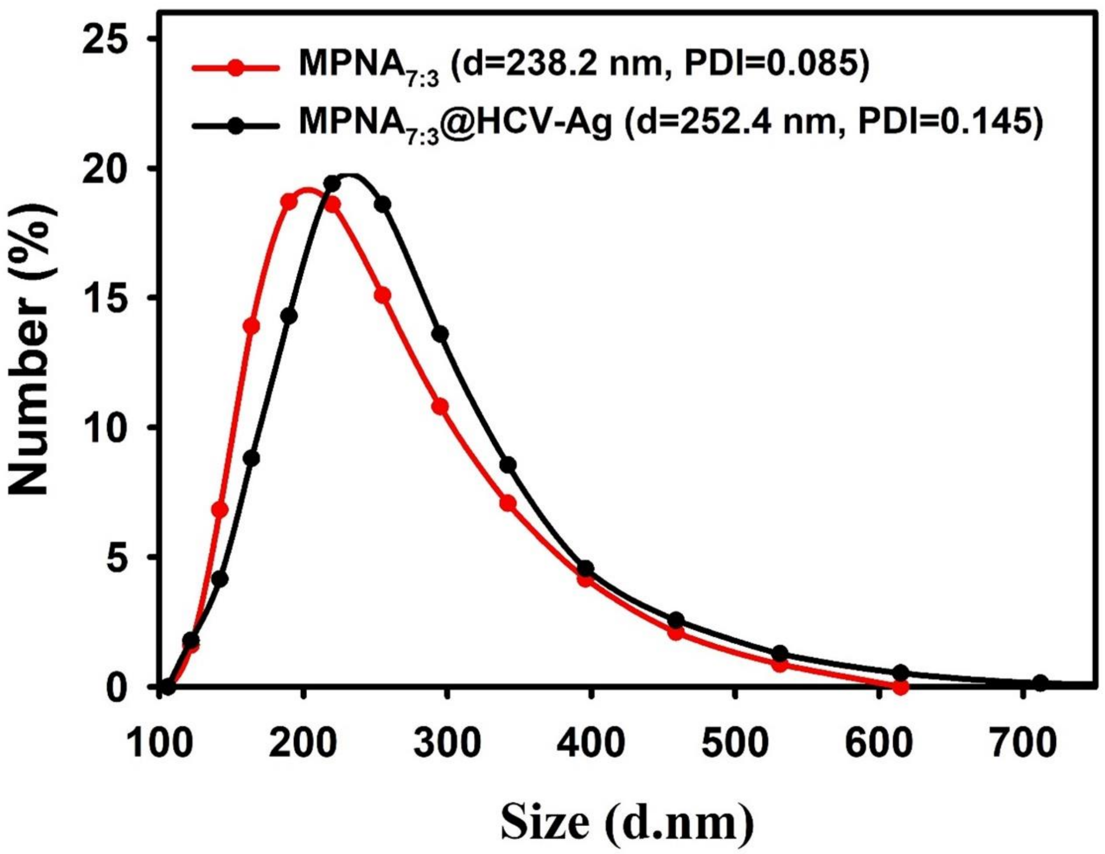

Figure S6. Hydrodynamic diameter distribution of $MPNA_{7:3}$ and $MPNA_{7:3}$@HCV-Ag.

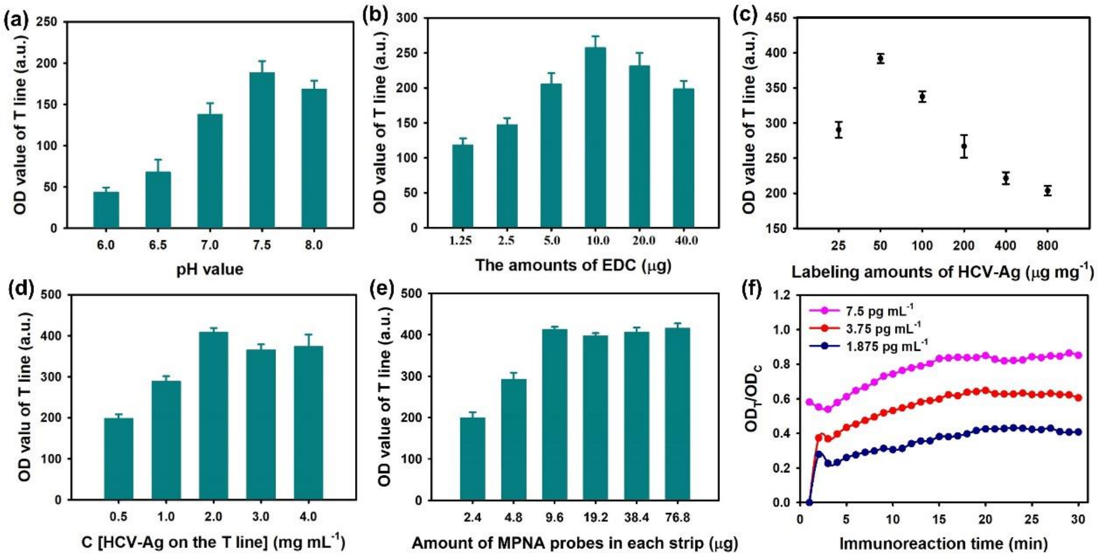

Figure S7. a-b) Effect of solution pH a) and the amounts of EDC b) in the process of coupling HCV-Ag on the surface of $MPNA_{7:3}$. c-e) Optimization of the amounts of HCV-Ag labeled to $MPNA_{7:3}$ c), the concentration of HCV-Ag sprayed into T line d) and the amounts of $MPNA_{7:3}$-HCV-Ag in each strip. e) Immunoreaction dynamics monitoring against reaction time and the concentration of anti-HCV.

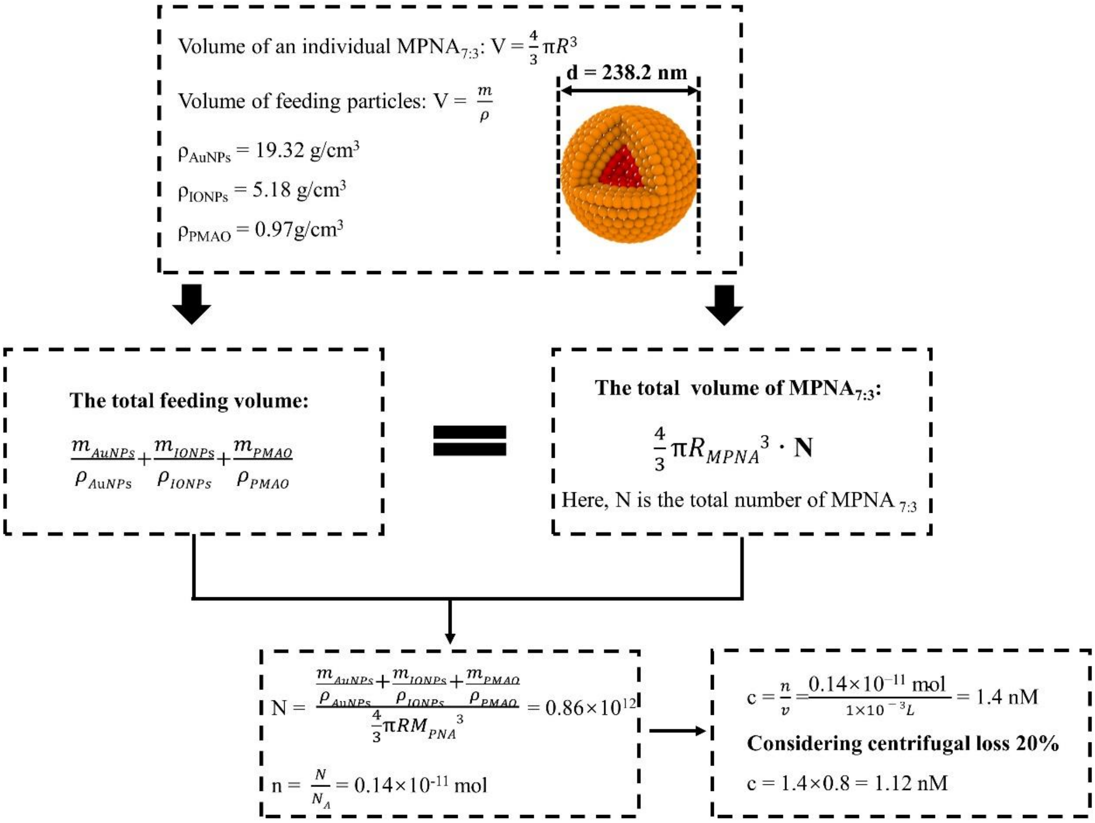

Figure S8. Calculation of the particle concentration of $MPNA_{7:3}$. The centrifugal loss was resulted via calculation the ratio of the absorbance of the supernatant after centrifugation to that before centrifugation.

Table S1. A summary of the synthesis condition of MPNAs with different feeding ratios of OA-AuNPs and OC-IONPs, as well as the corresponding plasmonic and magnetic performances of MPNAs at the same particle concentrations (15 pM).

The feeding ratios of OA-AuNPs/OC-IONPs	Hydrodynamic diameter (nm)	PDI	SPR peak (nm)	OD value	Magnetization（emu g^{-1}）	Magnetic recovery（%）
5:5	209.7	0.102	522	0.69	54.8	98.5
6:4	199.2	0.053	524	0.89	42.0	97.2
7:3	238.2	0.085	530	1.14	33.1	92.1
8:2	241.4	0.075	532	1.23	21.4	83.8
9:1	221.8	0.109	538	1.35	10.3	74.9

Table S2. Evaluation for the accuracy and precision of the $MPNA_{7:3}$-LFIA strip in human serum with five different anti-HCV concentrations.

Anti-HCV concentration (pg mL^{-1})	Intra-assay precision			Inter-assay precision[a]		
	mean[b]	CV (%)	Recovery (%)	mean[b]	CV (%)	Recovery (%)
60	1.40	9.43	107.80	1.39	7.28	104.77
30	1.21	5.95	102.00	1.23	2.15	111.34
15	1.00	6.02	90.43	1.01	10.34	92.42
7.5	0.84	1.63	95.92	0.86	7.67	104.32
3.8	0.66	4.18	95.65	0.64	5.90	90.47

[a] Assay was completed every one day for three days continuously.
[b] Mean value of four replicates at each diluted concentration.

Table S3. A correlation analysis for anti-HCV detection in ten clinical HCV-positive serum samples between the $MPNA_{7:3}$-LFIA strip and a commercial ELISA kit.

**Sample**	**$MPNA_{7:3}$-LIFA**		**Commercial ELISA kit**	
	**OD_T/OD_C**	**Anti-HCV amount measured (pg mL^{-1})**	**OD value**	**Anti-HCV amount measured (pg mL^{-1})**
1	0.569	2.54	0.021	3.04
2	1.452	78.55	1.154	95.1
3	0.645	3.41	0.036	4.23
4	1.389	61.49	0.858	71.04
5	0.798	6.18	0.046	5.06
6	1.268	38.42	0.368	31.25
7	0.925	10.13	0.170	15.1
8	0.617	3.06	0.010	2.15
9	0.915	9.74	0.085	8.22
10	0.892	8.91	0.072	7.15

Table S4. A correlation analysis for anti-HCV detection in 52 clinical serum samples between the $MPNA_{7:3}$-LFIA and TRIFMA method.

Serum sample	$MPNA_{7:3}$-LFIA (pg mL^{-1})	TRIFMA	Serum sample	$MPNA_{7:3}$-LFIA (pg mL^{-1})	TRIFMA	Serum sample	$MPNA_{7:3}$-LFIA (pg mL^{-1})	TRIFMA
1	+	+	19	+	+	37	—	—
2	+	+	20	+	+	38	—	—
3	+	+	21	+	+	39	—	—
4	+	+	22	+	+	40	—	—
5	+	+	23	+	+	41	—	—
6	+	+	24	+	+	42	—	—
7	+	+	25	+	—	43	—	—
8	+	+	26	+	—	44	—	—
9	+	+	27	+	—	45	—	—
10	+	+	28	+	—	46	—	—
11	+	+	29	+	—	47	—	—
12	+	+	30	+	—	48	—	—
13	+	+	31	+	—	49	—	—
14	+	+	32	+	—	50	—	—
15	+	+	33	—	—	51	—	—
16	+	+	34	—	—	52	—	—
17	+	+	35	—	—			
18	+	+	36	—	—			

Note: “+” and “—” represent positive and negative, respectively.

Table S5. The detailed concentrations of anti-HCV in 52 clinical serum samples detected using the $MPNA_{7:3}$-LFIA strip.

Serum sample	$MPNA_{7:3}$-LFIA (pg mL^{-1})	Serum sample	$MPNA_{7:3}$-LFIA (pg mL^{-1})	Serum sample	$MPNA_{7:3}$-LFIA (pg mL^{-1})
1	112.61	19	65.43	37	ND
2	85.18	20	44.10	38	ND
3	97.92	21	86.74	39	ND
4	104.36	22	22.28	40	ND
5	69.82	23	15.64	41	ND
6	21.55	24	105.10	42	ND
7	109.83	25	0.52	43	ND
8	55.47	26	3.62	44	ND
9	11.21	27	0.91	45	ND
10	8.90	28	0.78	46	ND
11	42.88	29	6.93	47	ND
12	22.63	30	2.41	48	ND
13	115.84	31	4.3	49	ND
14	23.90	32	0.3	50	ND
15	14.24	33	ND	51	ND
16	78.15	34	ND	52	ND
17	99.82	35	ND		
18	7.97	36	ND		

Note: ND represents "Not detected".
